# Four-year outcomes from an HIV research pathway program for aspiring scientists at the University of California, San Francisco Center for AIDS Research

**DOI:** 10.21203/rs.3.rs-10133790/v1

**Published:** 2026-07-08

**Authors:** Carina Marquez, Said Iftekhar Sadaat, Lauren Sterling, Joseph Watabe, Jonathan D. Fuchs, Audrey Paragan-Smith, Kelechi Uwaezuoke, Mallory O. Johnson, Monica Gandhi, John A. Sauceda

**Affiliations:** University of California, San Francisco; University of California, San Francisco; University of California, San Francisco; University of California, San Francisco; San Francisco Department of Public Health; San Francisco State University; San Francisco State University; University of California, San Francisco; University of California, San Francisco; University of California, San Francisco

## Abstract

**Background:**

Persistent inequities in the biomedical research workforce highlight the need for early, sustained training approaches that support individuals from backgrounds historically underrepresented in science and medicine. We present results of a 4-year evaluation of an HIV mentored research program for pre-doctoral students from colleges in the San Francisco Bay Area.

**Methods:**

The Center for AIDS Research (CFAR) Scholars Program took place at the University of California, San Francisco from 2022–2025. We conducted an evaluation across four cohorts of scholars (n = 23) before and after each year of the program. Scholars were individually mentored by a CFAR investigator, completed an independent research project, attended weekly didactic HIV seminars, and participated in professional development activities, along with clinical and community shadowing. We implemented pre- and post-program surveys to assess research skills, confidence, career clarity, program experiences, and mentorship quality each year. Qualitative data were collected through scholar focus groups and in-depth interviews with faculty mentors.

**Results:**

Across cohorts, scholars demonstrated consistent gains in research readiness, understanding of scientific processes, tolerance for research challenges, and clarity about their career paths. The proportion of scholars reporting high confidence in pursuing academic work increased from 52.2% to 86.4%, and interest in HIV research increased from 30.4% to 68.2%. All scholars reported a clear understanding of what a career scientist’s job entails, which was a significant improvement from baseline. Qualitative findings highlighted the central role of responsive and engaged mentorship, structured programming, and a strong sense of belonging within an HIV research community.

**Conclusions:**

Over four years, the UCSF CFAR Scholars Program showed sustained feasibility and a large impact in strengthening research skills, scientific identity, and HIV career interest among early-stage trainees. These findings support early mentored research pathways as an effective strategy to diversify and strengthen the HIV research workforce.

## Introduction

Persistent workforce inequities in academic medicine and biomedical research require new approaches to training the next generation of scientists and physician scientists. Despite decades of investment, first-generation students or those who are socioeconomically disadvantaged, have a disability, and/or are from racial and ethnic minority groups remain markedly less likely to enter, persist, and advance in academic research careers.^1–3^ Furthermore, failing to engage these students represent a missed opportunity to leverage untapped talent, particularly given the evidence that diverse teams in both business and science are more productive and creative.^4–6^

Within academia, later-stage or episodic training programs are insufficient to meaningfully diversify, secure, and retain the research pipeline. Instead, evidence points to the importance of early, sustained, and developmentally appropriate training experiences that build foundational research skills, scientific self-efficacy, professional identity, and provide access to high-quality mentorship.^7–10^ Prior work, including our own, shows that early mentored research experiences for undergraduate and masters-level trainees, can accelerate progression toward research careers by strengthening confidence, clarifying career trajectories, and supporting persistence in science.^11^

We previously described the implementation and first year-outcomes of a mentored HIV research training program for undergraduate and masters-level students at the University of California, San Francisco (UCSF) NIH-funded Center for AIDS Research (CFAR).^11^ In the inaugural cohort in 2022, we showed that a structured, paid, and applied research experience paired with intentional mentoring produced measurable gains in research skills, interest in HIV science, and identification as a scientist among trainees from underrepresented backgrounds. Importantly, this program was designed as a proof of concept, demonstrating what is possible with intensive intervention early in the academic pathway.

The UCSF CFAR Scholars was part of the CFAR Pathway Initiative,^12^ a national effort that sought to move from stand-alone mentoring programs to address a critical question: how can such programs be operationalized, scaled, and sustained across institutions and career stages. This initiative leveraged the longstanding CFAR network of United States-based, academically-affiliated HIV centers of excellence, as well as the CFAR network’s mission to accelerate HIV research and facilitate multidisciplinary collaboration. The Initiative included seventeen CFARs over its tenure and was designed to create linked, multilevel training pathways engaging scholars from high school through postdoctoral stages, in partnership with Historically Black Colleges and Universities and other Minority Serving Institutions. Across participating sites, programs integrated mentored research experiences, didactic training, professional development, and intentional community building, while allowing for local adaptation to institutional context and trainee needs.^13^

A single cross-site evaluation was done in year one of this national initiative, which demonstrated both feasibility and impact.^13^ Across the CFARs who published on their individual programs, scholars reported high satisfaction, increased interest in HIV research, and strong intentions to pursue further education and research careers.^14–16^ Qualitative findings underscored the central role of mentoring relationships, exposure to role models, and a sense of belonging within a research community.^13^ However, to date there are no reports on multi-year outcomes from any one of the CFAR Pathway Initiative programs. In this manuscript, we describe the four-year history of the UCSF CFAR Scholars Program and presents final pre-and post-program outcomes across four cohorts and longitudinal outcomes of scientific pursuits of the scholars in the program.

## Methods

### Enrolment.

Each summer, from 2022–2025, we selected six undergraduate and master’s students from a pool of applicants. Advertisements for the program included sending e-mails to Chairs and Deans of academic programs (e.g., psychology and biochemistry, student internship office) at local colleges and universities and promoting the program through flyers on social media and in-person and virtual tabling events at these institutions. Prospective applicants were required to submit copies of academic transcripts, two essays on career goals and interest in HIV research, a resume/CV, and a faculty recommendation. Applications were evaluated by the four members of the program’s leadership team and an alumnus of a similar training program in San Francisco. Selection of the scholars was based on a scoring system of how clearly and relevantly the applicants articulated their career goals, their prior research experience, the strength of the faculty recommendation, the quality of the resume or CV, academic performance, capacity to collaborate within diverse teams and any unique skills and lived experiences relevant to an applied research experience. Final selections were based on an “achievement relative to opportunity” criterion after scoring all sections of the application.

### Program Activities.

Each summer, we implemented a structured, paid mentored research experience. We paired each scholar with a UCSF HIV investigator based on research interests and availability of data for a clearly scoped, feasible research project to be completed over the summer months. Year 1 was a 16-week summer program, but this was shortened to 12 weeks in Year 2 and 3 after feedback from the scholars that it overlapped with the start of their fall school semester. In the final year of the program (Year 4–2025), because the funding mechanism for this program was removed by the NIH, we were forced to hold a 4-week program. To accommodate this shortened program in Year 4, scholars were hired at full-time (40 hours per week) rather than half-time (20 hours per week) as was done in Years 1–3. All other activities remained the same.

Program activities included weekly 1-on-1 mentor meetings; a structured weekly seminar series from investigators across the UCSF enterprise that covered the breadth of HIV science, research methods, and professional development (at least twice per week); cohort-based works-in-progress sessions with the two Co-Directors; and opportunities for clinical and community shadowing at the Ward 86 HIV Clinic at San Francisco General Hospital and local community-based health organizations. Scholars also participated in peer-learning activities, networking events, attended the International AIDS Conference virtually, and completed a “report back” presentation. Lastly, the program culminated in a final formal symposium where scholars presented their summer projects to the CFAR community at UCSF. Across cohorts, program leadership provided active coordination, mentor orientation and training, and ongoing evaluation to ensure consistency while allowing iterative refinement based on scholar and mentor feedback. Program activities are described in Table 1

### Program Evaluation.

We used quantitative and qualitative research methods to evaluate the program’s effectiveness each year and to identify areas for improvement. All qualitative evaluations, final reports, and recommendations for future programming were done independently by a consultant with expertise in program evaluation (Supplementary Content 1). The primary outcomes of our evaluation included:

1. *Assessing knowledge, skills, and confidence* – The 20-item Survey of Undergraduate Research Experience (SURE)^17^ measures the cognitive, personal, and professional development of scholars and was administered prior to starting the program and then again following the program. The survey items (e.g., *understanding of how scientists work on real problems, understanding of how knowledge is constructed, clarification of a career path*) were rated from 1 (*no gain or very small gain*) to 5 (*very large gain*). Gains were measured by comparing individual pre-post score differences from the scholars who completed the program.
2. *Assessing clarity on career path and interest in HIV research –* We assessed key aspects of scholars’ career plans. Response formats were based on the question format which focused on career clarity *(“when someone discusses a “career scientist,” how easy is it for you to visualize a career of that sort*?; “*To what extent would you say your career path is clear?*”), confidence (“*How would you rate your self-confidence to pursue academic work in the future?*”), and interest in HIV-related research (“*Rate your interest in pursuing a career that includes HIV research*?” in pre and post program surveys.
3. *Qualitative program evaluation*. The independent program evaluation conducted focus group discussions with the scholars and in-depth interviews with the faculty mentors after program completion. Over the four years of the program, four focus group discussions were conducted with scholars, with the participation of 5 scholars in each FGD. Twenty-three semi-structured individual in-depth interviews were conducted with faculty mentors. Transcripts from the focus group discussions and in-depth interviews were analysed utilizing a modified thematic analysis approach, which helped identify key codes, categories, and themes related to our program outcomes.

## Results

### Scholars Characteristics

Between 2022 and 2025, we enrolled a total 23 participants. In both the 2022 and 2023 cycles, we received 23 applications, interviewed 11 applicants, and admitted 6 scholars each year. One 2022 scholar took a medical leave of absence but rejoined to complete the program in 2023. In 2024, we received 22 applications, interviewed 12, and again admitted 6 scholars. One scholar suffered a medical injury outside of the program and could not return. In 2025, interest significantly increased with 74 applications, 10 interviews, and 6 admissions.

As shown in Table 2, the average age of scholars was 24.8 years, with 21 (91%) females, 1 male, and 1 declined to reveal gender. Of 23 participants enrolled, 12 (52.2%) identified as Latino/a/x, 4 (17.4%) as Black/African American, 3 (13.0%) as Western Asian, 2 (8.7%) as Middle Eastern, 1 (4.3%) as American Indian/Native Alaskan, and 1 (4.3%) as White non-Latino. Nearly half of the scholars (47.8%) were undergraduate students (i.e., 21.7% fourth-year, 17.4% third year, and 8.7% second-year undergraduate students), with 34.8% being master’s students and 17.4% post-baccalaureate students who had completed their undergraduate degrees.

### Quantitative Evaluation

**Figure 1:**
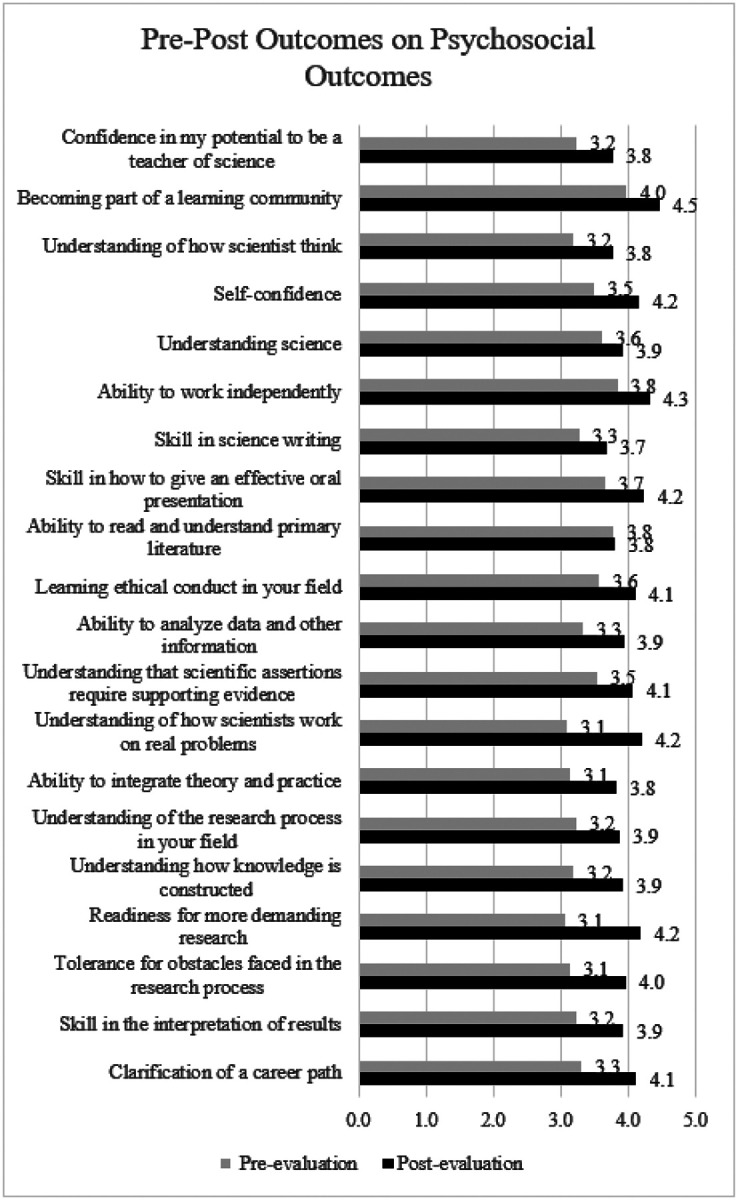
Survey Outcomes Among Four Cohorts of CFAR Scholars Program, 2022–2025. (N=23) **Note.**Pre-program response scores (gray bars) are defined as: 1 = No experience or feel inexperienced, 2 = Little experience, 3 = Some experience, 4 = Much experience, 5= Extensive experience or mastered this element. Post-program scores are defined as: 1 = No gain or very small gain, 2= Small gain, 3 = Moderate gain, 4 = Large gain, 5 = Extensive experience or mastered this element.

Pre-post program survey results are detailed in Fig. 1. The greatest improvements were among the following items [scale range 1–5]: 1. *readiness for more demanding research* (1.1 point gain), 2. *understanding of how scientists work on real problems* (1.1 point gain), 3. *tolerance for obstacles faced in the research process* (0.8 point gain), 4. *clarification of career path* (0.8 point gain), 5. *understanding how knowledge is constructed* (0.7 point gain). In total, from 2022 to 2025, on average across all 20 items, there was a 0.6 score improvement (i.e., from 3.4 to 4.0, or moderate to large gains).

#### Research identity and interest in HIV research.

Five scholars (22.7%) reported that they did not have any prior research experience before entering the program and 77.3% reported that they had some exposure to research (e.g., academic coursework or work in a research laboratory). In terms of HIV research, 18 (78.3%) of 23 reported that they had no or little experience with HIV research prior to the program. Critically, 21.7% reported that they did not have a clue or had a vague idea about how to visualize what a “career scientist” looked like. However, after the program, all scholars (100%) reported having a good or clear idea of what a “career scientist” looked like. Similarly, 86.4% of scholars reported *somewhat* or *very high self-confidence* in pursuing academic work in the future (up from 52.2%). Lastly, 68.2% reported *extensive interest* in pursuing a career in HIV research (up from 30.4% prior to the program).

#### Seminars, shadowing, and presentations.

Overall, our weekly seminar programming was deemed *highly valuable and very highly valuable* (scores of 4 and 5; range 1–5). These weekly HIV topics and methods seminars covered AIDS history, epidemiology of HIV, crafting research questions and hypotheses, current state of HIV prevention, treatment, and cure research, applied statistics, and scientific writing. Similarly, the clinical shadowing on HIV and infectious disease wards was also rated 100% valuable. A research scholar said, “I really enjoyed shadowing since it really shaped me in knowing what I wanted to do with my future.” The one-on-one weekly mentoring meetings were also rated *highly and very highly valuable* by 100% of the scholars.

#### Working with mentors.

On average, the scholars spent 5.2 hours per week meeting or working directly with their mentors. Almost all the scholars (95.5%) reported that the time they spent with their mentors was “just about right” rather than “not enough” or “too much.” Around 95.5% of the scholars reported that their mentor was about average or higher as a teacher and mentor. All the scholars consistently praised their mentors for being supportive, approachable, and encouraging. Overall, the mentorship experience was described as transformative, confidence-building, and deeply impactful on participants’ academic and career development. A scholar expressed, “My mentor was bubbly, structured, and really understood me culturally and as a person. He viewed me as a person and not as just another person they mentored.” Another scholar stated, “my mentor was very knowledgeable in his specialty in medicine, so it was helpful when asking him questions about clinical practice.”

#### Expectations of research project.

Most (82%) of the scholars *agreed* or *strongly agreed* that the scope and relevance of their research project or experience was appropriate for the summer period. Most (77%) scholars said that their research experience was *much better than expected*, and 100% of the scholars reported that they *felt good* about what they achieved for their research project.

#### Peer mentoring.

Most (72.7%) scholars reported that working with other students was one of the best parts of their research experience. All (100%) scholars were *satisfied* or *very satisfied* with the research experience with their peers.

#### Overall view of the program.

Approximately 95.5% of the scholars reported that they would recommend the CFAR UCSF Scholars Program to other students. The scholars provided open-ended responses indicating they understood the importance of networking, collaboration, and mentor-mentee dynamics. Overall, the program was described as empowering, informative, and deeply impactful.

### Qualitative Evaluation

Focus group data from the scholars describe overwhelmingly positive experiences and thoughtful recommendations for improving future programming (Table 3). The scholars noted the value of supportive mentorship they received and expressed that the existence of inbuilt HIV community and support networks that spanned across the CFAR team, mentors, investigative teams, and other staff positively impacted their experience of the program. The scholars also appreciated the importance of structured seminars and clinical shadowing, which deepened their understanding of HIV research. Additionally, scholars highlighted the significance of early goal setting with mentors, clearer communication channels, and tailored support for scholars conducting research.

Interview data from CFAR mentors also describe positive experience. Mentors reported being pleased and finding their participation in the program to be deeply rewarding, both professionally and personally. Many were inspired to mentor scholars to give back, having themselves benefited from strong mentorship within the CFAR and being from groups historically underrepresented in science and medicine. In Year 1, while they appreciated the structure and mission of the program, mentors expressed a desire for additional guidance on mentoring undergraduate students and orientation well in advance of onboarding their scholars, and more guidance on the scope of project designs and time management for short-term mentee engagements. This feedback was incorporated into the program in Years 2–4. Lastly, mentors emphasized the value of in-person interactions, mid-program evaluations, and more integrated support to ensure both mentors and scholars thrive.

## Discussion

This evaluation of the UCSF Center for AIDS Research Scholars Program provided robust quantitative and qualitative evidence that early mentored research engagement played a transformative role in cultivating a scientific identity, improving research skills, and enhancing HIV-related career interest among undergraduate and master’s students, especially those from underrepresented minority backgrounds. Over the course of four years, the program achieved high levels of scholar satisfaction, notable gains in research skills and confidence, and a measurable increase in scholars’ clarity about their academic and career trajectories.

The program also strengthened the scholars’ connections to careers in HIV research. Before the program, the scholars had limited or no exposure to HIV-related research, but at the end of the program, nearly all scholars reported a clearer vision of a scientist’s role, improved confidence in academic work, and heightened interest in pursuing HIV-related careers. A sister program, the Summer HIV/AIDS Research Program (SHARP),^10^ a 10 -week program implemented at the San Francisco Department of Public Health for undergraduates, observed similar outcomes, including employment in health research and matriculation into graduate degree and medical school programs.

The program’s mentorship model also emerged as a critical success factor. Scholars consistently praised their mentors for being supportive, friendly, culturally responsive, and intellectually engaged. Importantly, the one-on-one mentorship structure, combined with activities such as shadowing and project-based learning, facilitated both professional development and personal growth. Notably, the existing literature is robust regarding the value of mentorship in increasing the retention and success of underrepresented minority groups in science and medicine.^18,19^ This program applied many strategies to undergraduate and masters-level trainees from those mentorship models that focused on post-doctoral fellows and junior faculty; demonstrating that this structured and comprehensive mentoring can be applied to younger trainees.

While the program was well-received, scholars and mentors identified several areas for improvement. Critically, this evaluation was done independently, and the iterative nature of our evaluation allowed for prior year feedback to be incorporated into subsequent year programming. Moreover, given the short-term nature of these summer programs, which do offer the advantage of not competing with academic coursework, it is critical to develop guidance and offer support to help mentors and scholars craft and refine a feasible project that fits within the summer time frame and allows for the development of the scholars’ independent research questions and implementation of analysis. With intensive, structured effort, we found this to be possible as the program shortened to 12 weeks and even over the 4 weeks in the final year given the experiences we had gained over the four years.

### Evidence for Building and Refining Training Programs

Core features key to the program’s sustained success included paid, mentored research placements; clearly scoped, feasible projects; structured seminars on HIV science and research methods led by UCSF investigators; career development sessions on graduate and medical pathways; and opportunities for clinical and community exposure through shadowing. These features were reinforced by consistent one-on-one mentoring, cohort-based peer learning, and a strong emphasis on belonging within a research community.

We recommend that similar programs (1) align project scope and mentor expectations with the developmental stage and time constraints of early trainees; (2) pair applied research with structured, contextualized didactics rather than relying on immersion alone; (3) invest in mentor orientation and support to ensure clarity, responsiveness, and feasibility; (4) build flexibility into program delivery (e.g., hybrid options, scheduling accommodations) to reduce structural barriers to participation; and (5) provide financial support for both mentors and mentees. Perhaps not surprising, we did encounter scholars who worked second jobs during the program and on occasion forced scholars to miss some social events or shadowing opportunities. While we provided scholars with 50% support, other programs have successfully provided full-time employment. These features suggest that effective pathway programs are not defined by any single component, but by the intentional integration of mentoring, structure, evaluation, and adaptability to trainee needs. Most importantly, using holistic criteria, we selected and worked with scholars with little prior research experience, which yielded a high return on investment.

## Conclusion

This evaluation contributes to the growing evidence base supporting mentored research experiences as an effective strategy for increasing diversity in the biomedical sciences. The UCSF CFAR Scholars Program demonstrates that well-structured, equity-focused training programs can enhance research capacity, build confidence, and spark interest in HIV science among students from underrepresented backgrounds, ultimately strengthening the pipeline of diverse leaders in public health and academic research.

## Supplementary Material

This is a list of supplementary files associated with this preprint. Click to download.

- SupplmentaryContent.pdf

## Figures and Tables

**Table 1:** Overview of the UCSF Bay Area CFAR Scholars Program Activities

Mentoring and Independent Research Project	Core Curriculum	Program Evaluation and Capacity Building
**1-on-1 applied research and career mentorship** Mentors are HIV experts Mentor training from UCSF CFAR Mentoring Program Mentors compensated with salary or mentor training support Mentor-scholar matches based on research interest Mentors provided w/mentoring framework and contract to provide structure Individual Development Plan tailored to undergraduates. Goals, skills building vs. publications and grants Mentors meet with scholars weekly to guide them in a. identifying a research question, b. data collection (if applicable), c. data analysis, d. interpret findings, e. prepare for research symposium. Mentors review scholars career development goals CFAR letters of support for mentors for their advancement and promotion packets	**Core Curriculum**: Live and pre-recorded 16-week seminar program Min. of 2 seminars per week on methods and HIV topics (60 mins) plus 30 minutes for informal discussion from invited speaker and HIV expert (e.g., HIV and alcohol, HIV vaccine development). **Shadowing**: Placement in HIV and infectious disease inpatient teams and outpatient clinics, community-based research sites, basic and translational labs **Conference**: Virtual attendance at International AIDS Society Conference and scholar report back **Symposium and Graduation**: Formal presentation of research project/experience, integrated into the UCSF Bay Area CFAR Monthly Seminar series.	**UCSF and Collaborator Meetings**: Monthly meetings with consultants to review program design, progress, evaluation outcomes, synergizing of existing pathway programs and resources, and developing areas for future collaborations (Year 1). **Program Evaluation**: Part of qualitative evaluation by expert in pathway program development and valuation was formal report on areas of success and improvement for future cohorts.

**Table 2:** Characteristics of scholars and mentors for CFAR Scholars Program, 2022–2025 (N = 23)

Characteristics	N	%
**Age (years)**	24.8	
Under 25 years	10	43.5
25 years and over	13	56.5
**Enrollment year**		
2022	6	26.1
2023	5	21.7
2024	6	26.1
2025	6	26.1
**Gender**		
Female	21	91.3
Male	1	4.4
Undisclosed	1	4.3
**Education**		
Undergraduate student	11	47.8
Post-baccalaureate student	4	17.4
Master student	8	34.8
**Race/Ethnicity**		
Black/African American	4	17.4
American Indian/Native Alaskan	1	4.3
Latino	12	52.2
Western Asian	3	13
White non-Latino	1	4.4
Middle eastern	2	8.7

**Table 3:** Program Evaluation Summary of Findings (2022–2025).

Recommendations for to Improve Outreach/Recruitment, Application Process, Orientation	Recommendations to Improve Program Structure	Recommendations to Improve Research/Training Experience	Recommendations to Improve Mentorship Experience
2022			
Increase in-person meetings with mentors and other students for more humanizing effect of these interactions Provide an opportunity for scholars to become peer mentors for the current cohorts	Increase career clarity by having an opportunity to experience a “day in the life” of their mentors before joining the program. Encourage more organized social activities between scholars and mentors to foster deeper, more personal connections beyond academic work.	Increase opportunities for scholars to participate in data collection Create more structured group discussions and working sessions early in the program to help scholars collaboratively develop their research questions and build a sense of community	Encourage mentors to host “Safe Space” for communication with scholars
2023			
Expand outreach across campus departments for diversifying the application pool from which cohort was to be selected Assess mentors’ readiness and availability more carefully for early alignment in learning and teaching styles to ensure a successful mentor-scholar match.	Improve participation of scholars in program activities Offer more tailored workshops for early-stage scholars, such as premed/pre-health sessions	Promote scholar use of mentor data for projects and time in the field Improve scholars’ engagement, peer support, and community building by promoting more structured group work opportunities.	Expand the mentor selection process to enable scholars to meet or learn more about the mentors work prior to the matching process.-
2024			
Highlight and emphasize program restrictions/requirements during the interview and acceptance process Provide an internship agreement and require it to be reviewed periodically during the program	Provide a set calendar at the start of the program Provide a hybrid option for attending seminars and workshops Provide more flexible or increased time allotments for students doing lab bench work Offer a full-time stipend option to enhance to increase mentee integration into lab groups and so mentees do not need to get additional part time jobs	Encourage including mentees in relevant team or lab meetings to help them feel valued as team members and deepen their engagement with the research. Offer mentees experiences in field work and data collection when possible	Encourage mentors to establish clear expectations and communication preferences at the start of the mentoring relationship Implement a mentor ‘buddy system’ in the beginning of the program for mentors to exchange best practices in designing projects and integrating students in the lab. Offer additional training in mentoring undergraduates

## Data Availability

Data are available from the corresponding author upon reasonable request. Data with small cell sizes will be restricted to prevent participant re-identification.
